# Supplementary material for: Sorting at embryonic boundaries requires high heterotypic interfacial tension
Source: Nat Commun. 2017 Jul 31;8:157. doi: 10.1038/s41467-017-00146-x (PMC5537356; doi:10.1038/s41467-017-00146-x)
Supplement: Supplementary file 2 — Supplementary Software 1 [file 41467_2017_146_MOESM2_ESM.zip › PottsModel/SrcPottsModel/doc/model/SquarePositionManager.html]

SquarePositionManager


JavaScript is disabled on your browser.


Skip navigation links


- Overview
- Package
- Class
- Use
- Tree
- Deprecated
- Index
- Help

- Prev Class
- Next Class

- Frames
- No Frames

- All Classes

- Summary:
- Nested |
- Field |
- Constr |
- Method

- Detail:
- Field |
- Constr |
- Method


model

## Class SquarePositionManager

- java.lang.Object
- - model.SquarePositionManager

- All Implemented Interfaces:
  :   IPositionManager

  ---

    

  ```
  public class SquarePositionManager
  extends java.lang.Object
  implements IPositionManager
  ```

- - ### Nested Class Summary

    - ### Nested classes/interfaces inherited from interface model.IPositionManager

      `IPositionManager.Position`
  - ### Field Summary

    - ### Fields inherited from interface model.IPositionManager

      `HexagonPositions, SquarePositions`
  - ### Constructor Summary

    Constructors

    Constructor and Description

    `SquarePositionManager(Lattice pLattice)`
  - ### Method Summary

    All Methods Instance Methods Concrete Methods

    | Modifier and Type | Method and Description |
    | --- | --- |
    | `boolean` | `areHorizontalNeighbours(Pixel pPixel1, Pixel pPixel2)` |
    | `boolean` | `areNeighbours(Pixel pPixel1, Pixel pPixel2)` |
    | `boolean` | `areVerticalNeighbours(Pixel pPixel1, Pixel pPixel2)` |
    | `IPositionManager.Position[]` | `getAllNeighborPositions()` Returns left, bottom, right and top positions (in this order). |
    | `Pixel[]` | `getAllNeighbors(Pixel pPixel)` Returns an array of APixels whose length corresponds to the number of possible positions. |
    | `Pixel[]` | `getAllNeighbors(Pixel pPixel1, Pixel pPixel2)` Get union of neighbors of pPixel1 and pPixel2. |
    | `IPositionManager.Position[]` | `getConnectedNeighborPositions()` |
    | `Pixel[]` | `getConnectedNeighbors(Pixel pPixel)` |
    | `Pixel` | `getNeighborFromPosition(Pixel pPixel, IPositionManager.Position pPosition)` |
    | `IPositionManager.Position[]` | `getPartialNeighborPositions()` Returns partial neighbors so that iterating over all pixels and getting their respective partial neighbors will only result in passing over them a single time. |
    | `Pixel[]` | `getPartialNeighbors(Pixel pPixel)` |

    - ### Methods inherited from class java.lang.Object

      `equals, getClass, hashCode, notify, notifyAll, toString, wait, wait, wait`

- - ### Constructor Detail


    - #### SquarePositionManager

      ```
      public SquarePositionManager(Lattice pLattice)
      ```
  - ### Method Detail


    - #### getConnectedNeighborPositions

      ```
      public IPositionManager.Position[] getConnectedNeighborPositions()
      ```

      Specified by:
      :   `getConnectedNeighborPositions` in interface `IPositionManager`


    - #### getAllNeighborPositions

      ```
      public IPositionManager.Position[] getAllNeighborPositions()
      ```

      Returns left, bottom, right and top positions (in this order).

      Specified by:
      :   `getAllNeighborPositions` in interface `IPositionManager`

      Returns:


    - #### getPartialNeighborPositions

      ```
      public IPositionManager.Position[] getPartialNeighborPositions()
      ```

      Description copied from interface: `IPositionManager`

      Returns partial neighbors so that iterating over all pixels and getting their respective
      partial neighbors will only result in passing over them a single time. This is useful
      when computing the heterotypic boundary length for example and avoiding counting edges twice.

      Specified by:
      :   `getPartialNeighborPositions` in interface `IPositionManager`

      Returns:


    - #### getNeighborFromPosition

      ```
      public Pixel getNeighborFromPosition(Pixel pPixel,
                                           IPositionManager.Position pPosition)
      ```

      Specified by:
      :   `getNeighborFromPosition` in interface `IPositionManager`

      Returns:
      :   pPixel's neighbor from position pPosition or Null if the neighbor is out of bounds


    - #### getAllNeighbors

      ```
      public Pixel[] getAllNeighbors(Pixel pPixel)
      ```

      Description copied from interface: `IPositionManager`

      Returns an array of APixels whose length corresponds to the number of possible positions.
      However, some entries may be Null!

      Specified by:
      :   `getAllNeighbors` in interface `IPositionManager`

      Returns:


    - #### getConnectedNeighbors

      ```
      public Pixel[] getConnectedNeighbors(Pixel pPixel)
      ```

      Specified by:
      :   `getConnectedNeighbors` in interface `IPositionManager`


    - #### getPartialNeighbors

      ```
      public Pixel[] getPartialNeighbors(Pixel pPixel)
      ```

      Specified by:
      :   `getPartialNeighbors` in interface `IPositionManager`


    - #### getAllNeighbors

      ```
      public Pixel[] getAllNeighbors(Pixel pPixel1,
                                     Pixel pPixel2)
      ```

      Description copied from interface: `IPositionManager`

      Get union of neighbors of pPixel1 and pPixel2.

      Specified by:
      :   `getAllNeighbors` in interface `IPositionManager`

      Returns:


    - #### areNeighbours

      ```
      public boolean areNeighbours(Pixel pPixel1,
                                   Pixel pPixel2)
      ```

      Specified by:
      :   `areNeighbours` in interface `IPositionManager`


    - #### areHorizontalNeighbours

      ```
      public boolean areHorizontalNeighbours(Pixel pPixel1,
                                             Pixel pPixel2)
      ```

      Specified by:
      :   `areHorizontalNeighbours` in interface `IPositionManager`


    - #### areVerticalNeighbours

      ```
      public boolean areVerticalNeighbours(Pixel pPixel1,
                                           Pixel pPixel2)
      ```

      Specified by:
      :   `areVerticalNeighbours` in interface `IPositionManager`


Skip navigation links


- Overview
- Package
- Class
- Use
- Tree
- Deprecated
- Index
- Help

- Prev Class
- Next Class

- Frames
- No Frames

- All Classes

- Summary:
- Nested |
- Field |
- Constr |
- Method

- Detail:
- Field |
- Constr |
- Method
